# Recurrent Limitations of CAR-T Therapy in Gliomas: Evidence from Preclinical and Phase I Clinical Studies

**DOI:** 10.3390/ijms262311435

**Published:** 2025-11-26

**Authors:** Jessica Bria, Andrea Filardo, Anna Di Vito, Attilio Della Torre, Angelo Lavano, Isabella Coscarella, Emanuela Chiarella, Emanuela Procopio, Maria Teresa Egiziano, Prospero Longo, Domenico La Torre

**Affiliations:** 1 Department of Medical and Surgical Sciences, University Magna Graecia of Catanzaro, 88100 Catanzaro, Italy; jessica.bria@studenti.unicz.it (J.B.); filardo@unicz.it (A.F.); a.dellatorre@unicz.it (A.D.T.); lavano@unicz.it (A.L.); emanuelachiarella@unicz.it (E.C.); emanuela.procopio@studenti.unicz.it (E.P.); mariateresa.egiziano@studenti.unicz.it (M.T.E.); 2 Department of Clinical and Experimental Medicine, University Magna Graecia of Catanzaro, 88100 Catanzaro, Italy; divito@unicz.it (A.D.V.); isabella.coscarella@studenti.unicz.it (I.C.); 3 Department of Neurosurgery, Policlinico G. Martino, 98125 Messina, Italy; prospero.longo@studenti.unime.it

**Keywords:** CAR-T, CAR-T therapy, glioma, glioblastoma multiforme, molecular targets, organoids

## Abstract

In recent years, the development of new immunotherapy strategies has been a significant breakthrough in cancer treatment. Among these, engineered T cell therapy with chimeric antigen receptors (CAR-T) has produced notable clinical results, especially in hematological malignancies. This success has sparked growing interest in extending the application of CAR-Ts to solid tumors, including gliomas. Gliomas—in particular, glioblastoma multiforme (GBM)—are among the most aggressive primary brain tumors, associated with a poor prognosis and a median survival of approximately one year after diagnosis. However, the translation of CAR-T therapy to gliomas presents significant challenges, related to factors such as tumor heterogeneity, presence of the blood–brain barrier (BBB), and a strongly immunosuppressive tumor environment. Despite this, in recent years, there has been an intensification of research efforts aimed at the identification of new antigenic targets and the development of preclinical models—both in vitro and in vivo—to evaluate the efficacy and safety of CAR-Ts in the treatment of gliomas. Despite promising results, currently available models still have essential limitations in faithfully reproducing the complexity of human gliomas. This review aims to offer an exhaustive overview of the most recent preclinical studies on CAR-T therapy in gliomas, with a focus on the identification of molecular targets, experimental strategies aimed at overcoming immunological barriers, and translational challenges that need to be addressed for future successful clinical implementation.

## 1. Introduction

Gliomas are a heterogeneous group of primary tumors affecting the parenchyma of the central nervous system (CNS). They originate from glial cells and are among the most common and aggressive tumors in humans [1]. In 2021, the World Health Organization published the fifth edition of the classification of CNS tumors, integrating advances in understanding the molecular basis of gliomas with histopathology [2]. Despite advancements in treatment, a recent study by Zhang et al. (2025), which analyzed the period between 1990 and 2021, highlighted a 106% increase in cases and a 63.67% increase in mortality rate, confirming the highly lethal nature of these tumors and the persistent difficulties in improving therapeutic outcomes [3]. The 10% reduction in the age-standardized rate (ASR) of Disability-Adjusted Life Year (DALYs) is a positive sign, indicating improvements in clinical management and patient care, resulting in quality-of-life benefits; however, survival rates remain essentially stable, reflecting ongoing challenges in early diagnosis and the effectiveness of available treatments [3,4,5].

Despite the combined use of surgical resection, chemotherapy, and radiotherapy, supported by intraoperative neurophysiological monitoring, the prognosis remains poor, with a life expectancy of approximately 15 months [6,7]. This severe prognosis highlights the limitations of current therapeutic protocols. Further complicating this already critical clinical picture is the toxicity of standard glioma therapies, which is significantly influenced by several factors, including age, gender, and ethnicity. Age is the primary limiting factor, as elderly patients exhibit reduced tolerance to therapy and experience increased hematological toxicity (myelosuppression) due to a physiological decline in the bone marrow’s stem cell reserve [8,9]. Although the incidence of gliomas is higher in men, the toxic effects of temozolomide appear to affect women more. Recent studies have shown that women experience more severe and frequent thrombocytopenia and cytopenia than men [10,11]. Ethnicity and related pharmacogenetics can also influence patients’ toxicity profiles. A 2014 study by Bae et al. analyzed 300 Korean patients, suggesting that they may be more vulnerable to the gastrointestinal consequences of temozolomide than Western patients [12]. However, to date, data on different ethnicities remain limited, and further research is needed to improve our understanding of glioma biology in different populations. Indeed, a study by Dada et al., published in January 2025, highlighted that in a review of thirty-five studies, only three reported patient ethnicities; of those available, the data indicate that 91.1% of patients were white, 6.7% were black, and 2.2% were Hispanic [13].

In this context, a new cell therapy which has revolutionized the treatment of hematological cancers—namely, chimeric antigen receptor cell therapy (CAR-T)—is gradually gaining ground, with promising results even in the treatment of solid tumors such as GBM [14].

However, its application in solid tumors has encountered significant difficulties, such as crossing the BBB, which limits the passage of systemically administered CAR-T into the CNS.

Various local delivery strategies have been tested to overcome this obstacle and improve the intracerebral bioavailability of CAR-T. They have demonstrated promising results both in the preclinical setting and in early clinical trials [15,16].

Another limitation is a key characteristic of gliomas: the immunosuppressive tumor microenvironment (TME). Gliomas are recognized as “cold” tumors, characterized by tumor-infiltrating lymphocytes (TILs) with lower functional capabilities and a strong presence of immunosuppressive factors, such as transforming growth factor beta (TGF-β); interleukins such as IL-1, IL-2, IL-6, and IL-10; tumor necrosis factor alpha (TNF-α); and immunosuppressive cells. Liquid biopsy of the blood and cerebrospinal fluid (CSF) of glioblastoma patients confirmed the presence of these components [17]. Furthermore, the composition of the extracellular matrix (ECM) appears significantly altered in glioma [1]. Altogether, ECM composition within and around the tumor, immunosuppressive factors, and anatomical features such as the BBB strongly contribute to modulating CAR-T infiltration, trafficking, activation, and cytotoxicity.

A key feature of gliomas is the heterogeneity of tumor antigens, which is advantageous for immune escape. Indeed, several antigens, such as IL-13Rα2, EGFRvIII, and HER2, are expressed unevenly by tumor cells. This condition inevitably leads to the generation of antigen-negative cell clones after therapy. These phenomena are referred to as “antigen heterogeneity” and “immune escape” and are two of the main causes of tumor recurrence [16,18,19].

A limited number of clinical trials have been conducted to date, and most trials have investigated a small number of patients. Altogether, low enrollment in clinical trials, heterogeneous study designs, and the limited persistence and trafficking capacity of CAR-T cells, which are closely related to the immunosuppressive tumor microenvironment and the BBB, remain a crucial issue for the practical application of CAR-T therapy. Therefore, the aim of this study was to compare preclinical and clinical studies, focusing on the challenges associated with CAR-T therapy in glioma, and to identify possible strategies to increase clinical efficacy.

## 2. CAR-T Design, Cell Persistence, and Trafficking

### 2.1. Mechanism of Action of CAR-T Cells

CAR-T therapy involves genetically modifying a patient’s T cells to express chimeric antigen receptors (CARs) that recognize specific tumor-associated antigens. Once reinfused into the patient, these engineered T cells identify and eliminate tumor cells expressing the target antigen.

### 2.2. Structure of the CAR

The CAR is a synthetic fusion protein composed of three primary domains [14,20]:

- The extracellular domain, which is derived from a single-chain variable fragment (scFv) of an antibody. This fragment binds to a specific tumor antigen (e.g., EGFRvIII in gliomas or CD19 in lymphomas) in an MHC-independent manner [21].
- The transmembrane domain, whose purpose is to anchor the receptor to the T-cell membrane.
- The intracellular domain, which is responsible for transmitting the activation signal. The composition of the intracellular domain determines the generation of the CAR. To improve the efficacy and persistence of CAR-T cells after infusion, several modifications have been made to the intracellular domain, leading to the creation of five generations of CAR-T cells [22,23].

### 2.3. Activation, Cytotoxicity, and Persistence

**Figure 1:**
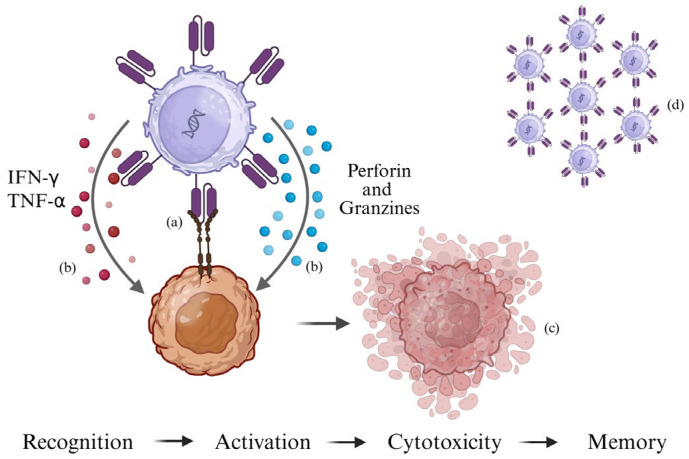
CAR-T cell recognizes the antigen on the tumor cell and activates (a). Through the release of perforins, granzymes, IFN-γ, and TNF-α (b), it induces tumor cell death (c). Following cytotoxic activity, a portion of the CAR-T cell persists as immunological memory (d) (created using https://BioRender.com).

Once the CAR binds to its target antigen, T lymphocytes are activated and trigger a cytotoxic response. CAR-T cells release cytokines such as interferon-gamma (IFN-γ) and TNF-α, which amplify immune activation, and cytotoxic molecules such as perforin and granzymes, which directly induce tumor cell death [24]. A distinctive feature of CAR-T cells is their ability to persist long-term once reintroduced into the patient, thus establishing an immunological memory that can protect against tumor recurrence. However, the duration of persistence is variable (Figure 1).

### 2.4. Production and Administration of CAR-T Cells

**Figure 2:**
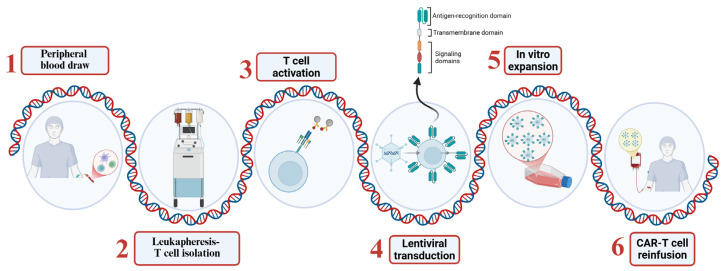
CAR-T therapy is personalized for each patient. T lymphocytes are isolated via leukapheresis from a peripheral blood sample from the patient. T cells are activated using magnetic beads coated with anti-CD3/CD28, and the T receptor is induced through lentiviral vector transduction. After transduction, CAR-T cells are expanded in vitro to obtain a therapeutic dose and then reinfused back into the patient. (Created in https://BioRender.com).

The production of CAR-T cells involves several steps:

- T cell isolation: T lymphocytes are collected from the patient’s peripheral blood through a process known as leukapheresis.
- Genetic modification: The isolated T cells are activated and genetically modified to express the CAR, typically through transduction with viral vectors such as lentiviruses or retroviruses [25].
- Expansion: Once the CAR-T cells have been genetically modified, they are expanded to obtain a therapeutic dose of cells.
- Reinfusion: Before CAR-T cell infusion, patients typically undergo lymphodepleting chemotherapy to facilitate engraftment [26]. The cells are then reinfused into the patient and begin to migrate to tumor sites (Figure 2).

The entire process typically takes 7–14 days from start to finish; however, next-generation CAR-T cell manufacturing strategies take 24–72 h [27].

### 2.5. Routes of Administration in Gliomas

The route of administration plays a significant role in the success rate of CAR-T therapy in glioma. Several strategies have been investigated, such as intravenous (IV) infusion, which is the most common and minimally invasive approach; intracranial locoregional administration, such as intratumoral (IT) and intracavitary (ICA) routes that use stereotactic catheters inserted directly into the tumor or resection cavity; intraventricular (ICV) administration into the cerebrospinal fluid (CSF) via the cerebral ventricles; and intrathecal (ITC) administration, with direct infusion into the subarachnoid space by lumbar puncture or catheter [15,19,28,29,30,31,32,33,34,35,36].

## 3. The Dilemma of CAR-T-Cell-Based Therapy in Glioma

In glioma, the therapeutic paradigm, known as Stupp’s Protocol, involves multimodal therapy combining the most extensive tumor surgery, followed by radiation therapy (RT) + Temozolomide (TMZ) and subsequent maintenance with TMZ [37]. However, the occurrence of post-treatment relapses highlights the urgency of exploring new therapeutic strategies such as CAR-T. The integration of novel strategies with standard therapies presents significant challenges. Indeed, an area of growing translational interest is the interaction of CAR-T with TMZ. The lymphotoxicity of TMZ compromises the expansion and persistence of infused CAR-T cells, making concomitant use problematic [38]. Several strategies to overcome this obstacle are being evaluated, one of which—now in the clinical phase—involves the use of TMZ in a lymphodepletion regimen to optimize the immunological environment before infusion [39]. Sampath et al. proposed a different approach, which has led to the creation of CAR-T cells engineered to be resistant to TMZ and directed against GBM IL-13Rα2+. Animals treated with combinations of resistant CAR-T cells and TMZ showed 2.8-fold-longer survival compared to those treated with sensitive CAR-T cells [38].

Despite encouraging progress, studies on CAR-T therapy in brain tumors are highly heterogeneous in terms of selection criteria, duration, and clinical endpoints assessed. This condition, combined with the limited number of patients enrolled in the studies, seriously hampers the comparability of the results and limits the possibility of drawing generalized conclusions [40] (Table 1).

### 3.1. Study Design: Small Cohorts and Limited Experimental Design

Most studies involve a few patients, often fewer than 10; for example, the study on anti-IL-13Rα2 CAR-T therapy included only one patient [15], while three patients were involved in the study of anti-HER2 CAR-T therapy [41], and four patients each were enrolled in Majzner’s and Barish’s studies [29,34]. Other studies have included slightly larger cohorts: those conducted by Brown and Bagley each included six patients [28,31], while O’Rourke’s study on anti-EGFR CAR-T cells enrolled 10 patients [19]. In a recent survey, Monje treated 11 patients with anti-GD2 CAR-T therapy [35]. An exceptional case is the 2024 study conducted by Brown, which evaluated CAR-T cells targeting IL-13Rα2 and included a larger cohort of approximately 65 patients, 58 of whom were evaluated for clinical response [30].

In many studies, the duration of follow-up is too short to effectively evaluate robust endpoints such as overall survival (OS) or progression-free survival (PFS). Early radiological endpoints are often used, but the objective response rate (ORR) remains generally low, often less than 10% [40]. Furthermore, the nature of the experimental design and phase I studies does not allow, in most cases, for comprehensive prognostic analyses or in-depth evaluations of individual cases [20]. In fact, many clinical studies focus mainly on the tolerability of CAR-T therapy, evaluating its immediate toxicity [30]. The correlation between immunological and biological responses to CAR-T therapy and patient prognosis is therefore complex [31]. These limitations underscore the need to develop nanomedicine platforms capable of exploiting nanoscale information, surpassing traditional imaging methods alone. In this context, the integration of single-molecule optical microscopy, which is capable of providing quantitative data on cellular mechanics, is a promising approach to further investigate the dynamics and nanomechanical variations in cells [42].

The use of Atomic Force Microscopy (AFM) with Infrared (IR) spectroscopy (AFM-IR), combined with unsupervised clustering and chemometrics, instead enables the identification of nanochemical and nanomechanical biomarkers, which are useful for disease monitoring and tailoring personalized therapeutic strategies. Finally, the creation of open-access data libraries containing experimental measurements and AFM settings is expected to promote the reproducibility of results and the development of artificial intelligence models capable of predictively interpreting mechanical and chemical variations at the nanoscale [43]. However, several inherent limitations of these techniques remain, including their low analytical throughput, operational complexity, and sensitivity to experimental parameters, which can compromise the reproducibility of results. Furthermore, poor penetration depth and the difficulty of analyzing heterogeneous or highly dynamic samples represent further obstacles to the routine use of AFM in the clinical setting [43,44].

### 3.2. Target Antigen Selection

The selection of target antigens in CAR-T cell therapy is a key factor affecting specificity, safety, and activation of the immune system. Preclinical and phase I clinical trials have identified several key targets, including (Table 2) those described below.

- IL-13Rα2: Overexpressed in over 50% of GBMs, with minimal expression in healthy brain tissue [16].
- EGFRvIII: A tumor-specific mutation; however, its heterogeneous expression contributes to antigen escape and tumor recurrence [19,21].
- HER2: Expressed in a subset of gliomas and other solid tumors, offering an additional target [18].
- GD2: Expressed by some gliomas, including glioblastomas [34,45,46].
- B7-H3: Expressed in a wide range of pediatric and adult solid tumors, with limited healthy tissue expression [47].
- ECM: A complex network composed of several multidomain macromolecules arranged in a tissue-specific manner, present in both normal and tumor tissues but differing in composition and function. The tumor ECM supports the aggressive biology of brain tumors, representing a potential strategy for GBM therapy [48].

#### 3.2.1. IL-13Rα2: A Selective Target for Glioblastoma

The IL-13Rα2 receptor is a high-affinity monomeric receptor for IL-13 that is overexpressed in more than 50% of GBMs and is associated with poor prognosis [32]. It is expressed in both stem-like and differentiated tumor cells, as well as in tumor-infiltrating macrophages and myeloid-derived suppressor cells. Notably, IL-13Rα2 is not significantly expressed in healthy brain tissue, making it a highly selective and safe target [49].

In an early clinical study, autologous CD8+ CTLs were engineered with first-generation IL13-zetakine CARs via DNA electroporation and ex vivo expansion. These cells demonstrated a median overall survival (OS) of 11 months. However, the small patient cohort (*n* = 3) significantly limited the clinical relevance and statistical power of the findings [32]. Subsequently, a second-generation CAR incorporating a point mutation (E12Y) and the 4-1BB costimulatory domain was developed, enabling preferential recognition of IL-13Rα2 over the ubiquitously expressed IL-13Rα1. In this study, 65 patients received at least one CAR-T infusion, and 58 were evaluated for clinical response [30]. Although safety and tolerability were confirmed, the overall survival remained comparable to previous studies, indicating limited clinical benefit [30,50].

#### 3.2.2. HER2: A Target for Midline and Hemispheric Pediatric Gliomas

HER2 is highly expressed in pediatric diffuse midline gliomas (DMGs)/DIPGs harboring H3 mutations, as well as in K27M wild-type and G34R-mutant hemispheric gliomas, making it a potential broad therapeutic target [51].

A recent clinical study by Vitanza et al. evaluated the locoregional delivery of balanced CD4:CD8 HER2-CAR-T cells in pediatric brain tumors. Although safety was demonstrated, the limited sample size (*n* = 3) and lack of long-term efficacy data were obstacles to broader clinical translation [36].

#### 3.2.3. EGFRvIII: A Relevant but Challenging Target

EGFRvIII is a constitutively active mutant of the epidermal growth factor receptor and is frequently expressed in GBMs. Despite its tumor specificity, its expression is highly heterogeneous and can be lost over time, severely limiting the durability and consistency of CAR-T responses [19]. This antigenic plasticity poses a significant risk of immune escape.

#### 3.2.4. GD2: An Emerging Target in Midline Gliomas

GD2 is a surface disialoganglioside highly expressed in DMGs, making it a promising immunotherapy target. Initial studies using lentiviral vectors to generate permanently expressed anti-GD2 CAR-T cells showed persistent expression of the CAR but were associated with localized inflammatory toxicity [52].

To address these safety concerns, more recent approaches have employed CAR-T cells based on transient mRNA, which have demonstrated dose-dependent anti-tumor efficacy and an improved safety profile in preclinical models. However, their limited persistence and the need for repeated administration are practical challenges for clinical application [52].

#### 3.2.5. B7-H3: A Promising Immunoregulatory Target with Limitations

B7-H3 (CD276) is an immune checkpoint ligand initially described as a T-cell costimulator but now recognized for its role in tumor progression, metastasis, and immune evasion. It is highly overexpressed in various solid tumors, including GBM, while showing minimal expression in healthy tissues [53,54].

Nevertheless, B7-H3 expression in GBM is heterogeneous, both between and within tumors. In vitro and in vivo studies have shown that CAR-T cell activity depends on high antigen density, meaning that tumors with low or patchy expression may escape immune targeting [55]. Furthermore, the fact that the in vivo study was conducted in a xenograft murine model limits its clinical translatability.

Another critical limitation is the suboptimal persistence of B7-H3 CAR-T cells, especially in models with high tumor burden. Although no significant off-tumor toxicity was reported, the presence of B7-H3 mRNA in healthy tissues raises concern for potential off-target effects, particularly in inflammatory conditions that could upregulate protein expression [56].

#### 3.2.6. The Extracellular Matrix: Beyond Structural Support

Recent studies have highlighted the importance of the ECM in glioma management. In tumors, the ECM shows profound differences in composition and architecture compared to that in normal tissue, generating a microenvironment that promotes tumorigenesis and metastasis [57]. ECM remodeling also plays a key role in immune targeting and drug resistance. However, although the targeting of ECM-related molecules such as CSPG4, BCAN, TNC, COL11A1, and GPC represents an innovative therapeutic strategy, current applications are still in the experimental phase and face several difficulties associated with the specificity, potential off-targets, and high heterogeneity of the ECM within the tumor [57].

Overall, based on these studies, target antigen selection remains one of the most important determinants of CAR-T therapy in gliomas. Evidence suggests that single-antigen approaches are insufficient to combat antigen evasion and tumor recurrence. Therefore, while the efforts and achievements made so far with this approach are commendable, future strategies should focus on multi-antigen therapy to overcome the limitations of heterogeneity, limit immune escape, and improve therapeutic durability.

**Table 2:** CAR-T cell therapy in gliomas: summary of targets, challenges, and limitations.

Target	Expression and Function in GBM	Clinical/ Preclinical Evidence	Key Limitations	Challenges/ Notes	Refs.
**IL-13Rα2**	Overexpressed in >50% of GBMs - Associated with poor diagnosis - Found in stem-like and differentiated tumor cells - Minimal expression in healthy brain	- First gen CAR-T: median OS ~11 months; - Second gen with E12Y + 4-1BB trial showed safety but limited benefit	- Limited clinical efficacy - Antigen escape with single-target CAR-T	- Development of multi-target strategies (e.g., IL-13Rα2 + B7-H3 or GD2/HER2)	[30,32,49]
**HER2**	- Highly expressed in pediatric DMGs and G34R-mutant gliomas - Also expressed in some GBMs and other solid tumors	- Pediatric trial locoregional delivery of balanced CD4:CD8 CAR-T showed safety but no strong efficacy data	- Very small sample size - Lack of long-term efficacy data	- Validation in larger cohorts - Demonstrated durable clinical benefit	[41]
**EGFRvIII**	- Tumor-specific but heterogeneously expressed	- Preclinical and early clinical studies showed feasibility and specificity	- High heterogeneity - Loss of antigen over time	- Strategies to overcome antigen escape - Combine with other targets	[19]
**GD2**	- Highly expressed in DMGs and some GBMs	- Lentiviral CAR-T: persistent expression but local inflammatory toxicity - mRNA CAR-T: dose-dependent anti-tumor effect; improved safety but transient persistence	- Toxicity in early studies - Short persistence with mRNA CAR-T	- Improve persistence while maintaining safety - Optimize dosing/repeat infusion schedules	[52]
**B7-H3 (CD276)**	- Overexpressed in GBMs and many solid tumors - Minimal expression in healthy tissue and heterogeneous within tumors	- Preclinical xenograft studies: tumor suppression with B7-H3 CAR-T - No major off-tumor toxicity observed	- Heterogeneous expression - Suboptimal persistence - Potential off-target effects if upregulated in inflamed tissues	- Enhance CAR-T persistence - Validate antigen density threshold -Humanized models	[55]
**ECM**	- Promotes tumor aggressiveness, immune evasion, and therapy resistance	- In vitro studies: targeting ECM molecules enhances T cell entry and boosts therapy	- Still in early stages - ECM present in both normal and tumor tissue	- Identify tumor-specific ECM components - Translate findings into clinical strategies	[57]

### 3.3. Tumor Microenvironment

The remodeling of the tumor microenvironment observed in gliomas represents one of the major strategies through which tumor cells hijack tissue components to support rapid cell proliferation, migration, and invasion, thus generating resistance. TME remodeling occurs at different levels through the recruitment and controlled polarization of cells, such as myeloid-derived suppressor cells (MDSCs), Tregs, TAMs, and microglia, or through the activation of specific pathways via the release of cytokines, extracellular vesicles, and growth factors which are able to mediate cell-to-cell interactions [58,59,60]. In gliomas, MDSCs are polarized to promote tumorigenesis and immunosuppression. Similarly, Tregs—attracted by the TME and various chemokines through cell–cell contact—exert immunosuppressive effects, establishing a functional synergy with MDSCs [61]. Macrophages also migrate to the tumor site, where tumor cells are responsible for a switch in macrophage phenotype, favoring a pro-invasive and immunosuppressive M2 state via the release of immunosuppressive factors, such as CSF-1, CCL2, IL-4, IL-6, IL-10, and TGF-β [58].

Furthermore, cell interactions involve not only tumor cells and immune cells but also endothelial cells, neurons, glia, oligodendrocytes, and ECM proteins (hyaluronic acid, collagen, etc.) [62].

Therefore, glioma cell-guided TME reprogramming depends on complex cell–cell and cell–matrix interactions. This scenario, further influenced by increased ECM stiffness and chemical components such as low oxygen availability and low pH, obstructs CAR-T cell infiltration, contributing to the exhaustion and dysfunction of CAR-T cells [33,63].

Numerous strategies have been developed to produce CAR-T cells with enhanced anti-tumor function [64], among which the addition to culture media of specific cytokines, metabolic modulators, antioxidants, epigenetic modifiers, and pharmacological inhibitors targeting specific signaling pathways has shown significant improvements in the persistence and long-term effectiveness of CAR-T cells in solid tumors. In recent years, tools have been developed to recreate the complexity of the TME. These devices, known as Tumors-on-a-Chip (ToCs), can apply controlled perfusion by mimicking the mechanical forces of cellular flows, such as interstitial flow (IF) [65]. These forces, in addition to transporting nutrients and metabolites, act as mechanobiological signals that modulate intracellular signaling pathways. The combination of spheroids/organoids with microfluidic technology is of particular interest for understanding tumor biology and accelerating the development of new therapies [66]. However, further research is required to ensure CAR-T cell function in the TME.

### 3.4. Delivery and Access

The administration of CAR-T cells to the tumor “situ” represents a real challenge for the treatment of gliomas. Different routes of administration have been tested in various clinical trials, including IV, ICA, and ICV injections [19,28,29,30,32,35,36,47,67].

The choice of the route of administration itself represents a crucial first step. Although the IV route is minimally invasive compared to the ICA and ICV routes, it is related to severe side effects, including severe Immune Effector Cell-Associated Neurotoxicity Syndrome (ICANS) and Cytokine Release Syndrome (CRS), especially at higher doses [19,35,47].

On the other hand, both the ICA and IT routes of administration are well tolerated. However, especially when combined, they can have serious neurological consequences, such as encephalopathy, ataxia, hemiparesis, hydrocephalus (all in dual ITC/ICV), and cerebral edema with a g3 > 35% toxicity [15,29,30,31,32,67].

The ICV pathway offers direct access to the CSF system, bypassing the BBB. However, its high invasiveness requires complex management of the patient and incurs a high risk of adverse events, including encephalopathy, hypertension, ICANS, and Tumor Inflammation-Associated Neurotoxicity (TIAN) [30,35,47].

To date, there is no agreement on which is the best route of administration, indicating the need to evaluate each case individually.

### 3.5. Toxicity

Although the management of the patient post-CAR-T infusion is a well-known critical issue due to persistent neurological or inflammatory complications, such as CRS, ICANS, and TIAN, the analysis of demographic factors in relation to the specific toxicity of CAR-T therapy is a recent area of research, with data yet to be consolidated. The currently available evidence seems to suggest that gender and race are not significant predictors of the onset and severity of CAR-T toxicity [68]. Age, however, remains a relevant factor; elderly patients, especially those with comorbidities, are more susceptible to CRS and ICANS, complicating the clinical management of adverse reactions [69].

#### 3.5.1. Cytokine Release Syndrome (CRS)

Cytokine Release Syndrome is a systemic inflammatory response triggered by the rapid and massive release of cytokines from activated immune cells, including CAR-T cells. This syndrome is a well-documented and potentially severe side effect of CAR-T therapy, manifesting with a wide range of constitutional and organ-specific symptoms [15,19,28,29,30,32,35,47,67,70,71]. CRS is the most frequent systemic adverse event occurring in patients. The symptomatology of CRS typically includes fever, chills, malaise, headaches, myalgias, arthralgias, and anorexia. Severe CRS can progress to hypotension, hypoxia, and multi-organ failure, necessitating prompt recognition and intervention [15,19,28,29,30,32,35,47,67,70,71].

#### 3.5.2. Immune Effector Cell-Associated Neurotoxicity Syndrome (ICANS)

Immune Effector Cell-Associated Neurotoxicity Syndrome is an acute neurotoxicity syndrome that affects 15–30% of patients, who present with symptoms such as aphasia, attention deficit, and seizures. The prevalence of neurological adverse events (headache, seizures, and encephalopathy) in clinical trials of CAR-T for gliomas is not simply the result of general CAR-T therapy toxicity. Still, it is potentially exacerbated or occurs solely due to the intrinsic sensitivity of the brain and the CNS-directed administration routes often used for gliomas [72]. It results from immune hyperactivation that leads to disruption of the BBB and cytokine infiltration into the CNS. Diagnosis is based on clinical assessment supported by neuroimaging and CSF analysis [19,28,30,35,36,47,67,70].

#### 3.5.3. Tumor Inflammation-Associated Neurotoxicity (TIAN)

Recently identified in patients with CNS tumors, unlike ICANS, TIAN is a neuroinflammatory reaction localized at the tumor site. It manifests as a transient exacerbation of pre-existing focal neurological deficits and/or peritumoral edema visible on MRI. Treatment typically involves corticosteroids and diuretics. TIAN has been classified into two types:

- Type 1—mechanical effects caused by edema, such as increased intracranial pressure or hydrocephalus, requiring urgent intervention [73];
- Type 2—characterized by transient neural circuit dysfunction and self-limiting. Notably, all reported patients have fully recovered, with no TIAN-related deaths [35,47,73].

## 4. Future Perspectives

### 4.1. Multitargeting CAR-T Cells: A Strategy Against Tumor Heterogeneity

To counteract the cellular heterogeneity that contributes significantly to disease recurrence and therapeutic resistance in GBM, Meghan Logun et al. have developed a bivalent CAR construct capable of targeting both the epidermal growth factor receptor (EGFR) and IL-13Rα2 [74]. In their study, patients first underwent surgical reduction of recurrent tumor, during which an Ommaya reservoir was implanted to enable ITC administration of CAR-T cells. After recovery, the patients received a single dose of CAR-T cells 1 × 10^7^ cells (*n* = 3) and 2.5 × 10^7^ cells (*n* = 3) of bivalent EGFR–IL-13Rα2 intrathecally. An interim analysis of the first six patients confirmed the initial feasibility and tolerability of this approach [74]. A similar study conducted by Bagley et al. confirmed tolerability and toxicity; however, treatment was associated with early-onset neurotoxicity, consistent with immune effector cell-associated neurotoxicity syndrome (ICANS), which was managed with high-dose dexamethasone and anakinra (an IL-1 receptor antagonist). In particular, a patient at the highest dosage experienced dose-limiting toxicity (grade 3 anorexia, generalized muscle weakness, and fatigue) [28]. Both studies, from the point of view of effectiveness, highlight an early reduction in tumor size and contrastographic enhancement on MRI, an indicator of tumor activity and vascular permeability [28,74].

### 4.2. Patient-Derived Organoids

Given this complex network of local and systemic immunosuppressive mechanisms, there is a growing need for preclinical models that accurately reflect GBM tumor biology and its interaction with the immune system. In this context, patient-derived organoids (PDOs) are emerging as promising tools for studying the tumor microenvironment in a personalized manner. By preserving the genetic and phenotypic heterogeneity of the original tumor—and, in some cases, its immune components—PDOs provide an ideal platform for testing immuno-oncology therapies and evaluating individual treatment responses in vitro [75].

Brain organoids are three-dimensional cultures derived from stem cells that self-organize and differentiate to mimic key structural and functional features of the human brain. In glioblastoma research, patient-derived tumor organoids preserve tumor heterogeneity and the native microenvironment, providing a more physiologically relevant preclinical model than traditional 2D systems. These organoids offer an intermediate platform between in vitro and in vivo studies, supporting both biological investigation and the development of personalized therapies [75,76].

Building upon this approach, Amanda Linkous et al. developed a sophisticated model by co-culturing GFP-labeled glioma stem cells (GSCs) with brain organoids derived from human embryonic stem cells (hESCs) or induced pluripotent stem cells (iPSCs). Their cerebral organoids replicate key developmental brain structures, including ventricular zones and neural stem cell populations marked by Nestin, Sox2, Pax6, and TBR2 [77].

Following one week of co-culture, the GSCs infiltrate and proliferate within the organoids, accurately recapitulating the invasive growth patterns and histopathological features of human glioblastoma tumors [75].

Brain organoids represent an advanced model to study glioblastoma in a three-dimensional context that reflects tumor complexity. Separately, it has been shown that chimeric antigen receptor T (CAR-T) cells can be co-cultured with patient-derived glioblastoma organoids (GBOs) to evaluate their efficacy and specificity. In this system, 2173 CAR-T cells targeting EGFRvIII+ cells specifically killed target cells while sparing EGFRvIII- cells [78].

## 5. Conclusions and Future Perspectives

Current phase I clinical trials and preclinical studies of CAR-T cell therapy in gliomas point to several critical limitations that hinder the efficacy and clinical translatability of this therapeutic strategy. Small patient groups and heterogeneous study designs reduce statistical power and complicate inter-study comparisons, underlining the need for larger, standardized studies [15,41]. The limited persistence and trafficking capacity of CAR-T cells—mainly due to the immunosuppressive tumor microenvironment and the BBB—reduce the duration and penetration of the therapeutic effect [19,34]. Tumor heterogeneity and the antigen escape phenomenon are further significant obstacles, highlighting the need for multitarget strategies and combination therapies to overcome resistance [74,79]. Furthermore, the absence of pre-infusion lymphodepletion regimens and the uncontrolled use of concomitant drugs may negatively influence CAR-T activity [40]. Finally, insufficient follow-up duration limits the possibility of evaluating long-term outcomes, while low objective response rates indicate the need for more adequate clinical endpoints [34,41].

Overcoming these limitations will require innovative preclinical models that better reproduce the complexity of human glioma, such as patient-derived organoids and biobanks with detailed clinical data [80], and more rigorous and harmonized clinical experimental designs. By focusing on these improvements, future research can increase the effectiveness of CAR-T therapy and accelerate its translation into effective treatments for glioma patients.

Based on this evidence, future research activities should address current limitations through innovative strategies, such as

- Multi-targeted CARs, designed to reduce antigen escape by targeting multiple tumor antigens simultaneously;
- “Armored” CARs, designed to resist the immunosuppressive tumor microenvironment; for example, by secreting immunostimulatory cytokines such as IL-12 [81];
- Combination therapies, encompassing the use of CAR-T cells in combination with immune checkpoint inhibitors or radiotherapy to improve their efficacy;
- “Standard” CAR-T cells, encompassing the development of allogeneic CAR-T cells from healthy donors to reduce production times and costs [25].

## Figures and Tables

**Table 1:** Classification of obstacles in CAR-T therapy of gliomas.

Category	Limitations
Study Design	- Early-phase, low-powered trials - Lack of biomarker-driven patient selection - Clinical endpoints and follow-up duration
Target antigen selection	- Single antigen CAR-T therapies can promote antigen escape, making them ineffective
Tumor Microenvironment (TME)	- Profound immunosuppression (Tregs, MDSCs, TAMs) - Hypoxia and acidic pH - Barrier to CAR-T infiltration
Delivery and Access	- Blood–brain barrier (BBB) limits systemic CAR-T entry - Locoregional (Ommaya/intracerebral) preferred
Toxicity	- Neurotoxicity - Potential for on-target/off-tumor effects

## Data Availability

Not applicable.

## References

[1] Di Vito A., Donato A., Bria J., Conforti F., La Torre D., Malara N., Donato G. (2024). Extracellular Matrix Structure and Interaction with Immune Cells in Adult Astrocytic Tumors. Cell. Mol. Neurobiol..

[2] Berger T.R., Wen P.Y., Lang-Orsini M., Chukwueke U.N. (2022). World Health Organization 2021 Classification of Central Nervous System Tumors and Implications for Therapy for Adult-Type Gliomas: A Review. JAMA Oncol..

[3] Zhang Q., Yu H., Zhong J., Cheng W., Qi Y. (2025). Global, Regional, and National Burden of Brain and Central Nervous System Cancer: A Systematic Analysis of Incidence, Deaths, and DALYS with Predictions to 2040. Int. J. Surg..

[4] Kocarnik J.M., Compton K., Dean F.E., Fu W., Gaw B.L., Harvey J.D., Henrikson H.J., Lu D., Pennini A., Global Burden of Disease 2019 Cancer Collaboration (2022). Cancer Incidence, Mortality, Years of Life Lost, Years Lived with Disability, and Disability-Adjusted Life Years for 29 Cancer Groups From 2010 to 2019: A Systematic Analysis for the Global Burden of Disease Study 2019. JAMA Oncol..

[5] GBD 2016 Brain and Other CNS Cancer Collaborators (2019). Global, Regional, and National Burden of Brain and Other CNS Cancer, 1990–2016: A Systematic Analysis for the Global Burden of Disease Study 2016. Lancet Neurol..

[6] Guzzi G., Ricciuti R.A., Della Torre A., Lo Turco E., Lavano A., Longhini F., La Torre D. (2024). Intraoperative Neurophysiological Monitoring in Neurosurgery. J. Clin. Med..

[7] Torregrossa F., Aguennouz M., La Torre D., Sfacteria A., Grasso G. (2019). Role of Erythropoietin in Cerebral Glioma: An Innovative Target in Neuro-Oncology. World Neurosurg..

[8] Saito K., Mukasa A., Narita Y., Tabei Y., Shinoura N., Shibui S., Saito N. (2014). Toxicity and Outcome of Radiotherapy with Concomitant and Adjuvant Temozolomide in Elderly Patients with Glioblastoma: A Retrospective Study. Neurol. Med. Chir..

[9] Tao Q., Zhu T., Ge X., Gong S., Guo J. (2022). The Efficacy and Safety of Low-Dose Temozolomide Maintenance Therapy in Elderly Patients with Glioblastoma: A Retrospective Cohort Study. Ann. Palliat. Med..

[10] van Hout L., Borgo A.D., Grun N., Schuur M., Broen M.P.G., Westerman B.A., Bartelink I., Vandertop W.P., Lissenberg-Witte B.I., Kouwenhoven M.C.M. (2025). Severe Temozolomide-Induced Thrombocytopenia Is Linked to Increased Healthcare Utilization in Glioblastoma and Disproportionally Impacts Female Patients. Neurooncol. Pract..

[11] Zeiner P.S., Filipski K., Filmann N., Forster M.-T., Voss M., Fokas E., Herrlinger U., Harter P.N., Steinbach J.P., Ronellenfitsch M.W. (2022). Sex-Dependent Analysis of Temozolomide-Induced Myelosuppression and Effects on Survival in a Large Real-Life Cohort of Patients with Glioma. Neurology.

[12] Bae S.H., Park M.-J., Lee M.M., Kim T.M., Lee S.-H., Cho S.Y., Kim Y.-H., Kim Y.J., Park C.-K., Kim C.-Y. (2014). Toxicity Profile of Temozolomide in the Treatment of 300 Malignant Glioma Patients in Korea. J. Korean Med. Sci..

[13] Dada O.E., Chisango Z., Nkansah-Poku K.A.B., Sowah M.N., Rodrigues A.C.L.F., Duru O., Myers M., Williams S.T., Ushewokunze S., Collis S.J. (2025). Race and “Omic” Data in Glioma: A Systematic Review of Contemporary Research to Explore the Digital Divide. Neuro-Oncol. Pract..

[14] June C.H., Sadelain M. (2018). Chimeric Antigen Receptor Therapy. N. Engl. J. Med..

[15] Brown C.E., Alizadeh D., Starr R., Weng L., Wagner J.R., Naranjo A., Ostberg J.R., Blanchard M.S., Kilpatrick J., Simpson J. (2016). Regression of Glioblastoma after Chimeric Antigen Receptor T-Cell Therapy. N. Engl. J. Med..

[16] Feldman L., Brown C., Badie B. (2022). Chimeric Antigen Receptor (CAR) T Cell Therapy for Glioblastoma. Neuromol. Med..

[17] Coluccio M.L., Presta I., Greco M., Gervasi R., La Torre D., Renne M., Voci C.P., Lunelli L., Donato G., Malara N. (2020). Microenvironment Molecular Profile Combining Glycation Adducts and Cytokines Patterns on Secretome of Short-Term Blood-Derived Cultures during Tumour Progression. Int. J. Mol. Sci..

[18] Hegde M., Mukherjee M., Grada Z., Pignata A., Landi D., Navai S.A., Wakefield A., Fousek K., Bielamowicz K., Chow K.K.H. (2016). Tandem CAR T Cells Targeting HER2 and IL13Rα2 Mitigate Tumor Antigen Escape. J. Clin. Investig..

[19] O’Rourke D.M., Nasrallah M.P., Desai A., Melenhorst J.J., Mansfield K., Morrissette J.J.D., Martinez-Lage M., Brem S., Maloney E., Shen A. (2017). A Single Dose of Peripherally Infused EGFRvIII-Directed CAR T Cells Mediates Antigen Loss and Induces Adaptive Resistance in Patients with Recurrent Glioblastoma. Sci. Transl. Med..

[20] June C.H., O’Connor R.S., Kawalekar O.U., Ghassemi S., Milone M.C. (2018). CAR T Cell Immunotherapy for Human Cancer. Science.

[21] Maude S.L., Frey N., Shaw P.A., Aplenc R., Barrett D.M., Bunin N.J., Chew A., Gonzalez V.E., Zheng Z., Lacey S.F. (2014). Chimeric Antigen Receptor T Cells for Sustained Remissions in Leukemia. N. Engl. J. Med..

[22] Sadelain M., Brentjens R., Rivière I. (2013). The Basic Principles of Chimeric Antigen Receptor Design. Cancer Discov..

[23] Ramesh P., Hui H.Y.L., Brownrigg L.M., Fuller K.A., Erber W.N. (2023). Chimeric Antigen Receptor T-Cells: Properties, Production, and Quality Control. Int. J. Lab. Hematol..

[24] Porter D.L., Levine B.L., Kalos M., Bagg A., June C.H. (2011). Chimeric Antigen Receptor-Modified T Cells in Chronic Lymphoid Leukemia. N. Engl. J. Med..

[25] Depil S., Duchateau P., Grupp S.A., Mufti G., Poirot L. (2020). “Off-the-Shelf” Allogeneic CAR T Cells: Development and Challenges. Nat. Rev. Drug Discov..

[26] Turtle C.J., Hanafi L.-A., Berger C., Gooley T.A., Cherian S., Hudecek M., Sommermeyer D., Melville K., Pender B., Budiarto T.M. (2016). CD19 CAR-T Cells of Defined CD4+:CD8+ Composition in Adult B Cell ALL Patients. J. Clin. Investig..

[27] Ayala Ceja M., Khericha M., Harris C.M., Puig-Saus C., Chen Y.Y. (2024). CAR-T Cell Manufacturing: Major Process Parameters and next-Generation Strategies. J. Exp. Med..

[28] Bagley S.J., Logun M., Fraietta J.A., Wang X., Desai A.S., Bagley L.J., Nabavizadeh A., Jarocha D., Martins R., Maloney E. (2024). Intrathecal Bivalent CAR T Cells Targeting EGFR and IL13Rα2 in Recurrent Glioblastoma: Phase 1 Trial Interim Results. Nat. Med..

[29] Barish M.E., Aftabizadeh M., Hibbard J., Blanchard M.S., Ostberg J.R., Wagner J.R., Manchanda M., Paul J., Stiller T., Aguilar B. (2025). Chlorotoxin-Directed CAR T Cell Therapy for Recurrent Glioblastoma: Interim Clinical Experience Demonstrating Feasibility and Safety. Cell Rep. Med..

[30] Brown C.E., Hibbard J.C., Alizadeh D., Blanchard M.S., Natri H.M., Wang D., Ostberg J.R., Aguilar B., Wagner J.R., Paul J.A. (2024). Locoregional Delivery of IL-13Rα2-Targeting CAR-T Cells in Recurrent High-Grade Glioma: A Phase 1 Trial. Nat. Med..

[31] Brown C.E., Rodriguez A., Palmer J., Ostberg J.R., Naranjo A., Wagner J.R., Aguilar B., Starr R., Weng L., Synold T.W. (2022). Off-the-Shelf, Steroid-Resistant, IL13Rα2-Specific CAR T Cells for Treatment of Glioblastoma. Neuro-Oncology.

[32] Brown C.E., Badie B., Barish M.E., Weng L., Ostberg J.R., Chang W.-C., Naranjo A., Starr R., Wagner J., Wright C. (2015). Bioactivity and Safety of IL13Rα2-Redirected Chimeric Antigen Receptor CD8+ T Cells in Patients with Recurrent Glioblastoma. Clin. Cancer Res..

[33] Liu G., Rui W., Zhao X., Lin X. (2021). Enhancing CAR-T Cell Efficacy in Solid Tumors by Targeting the Tumor Microenvironment. Cell. Mol. Immunol..

[34] Majzner R.G., Ramakrishna S., Yeom K.W., Patel S., Chinnasamy H., Schultz L.M., Richards R.M., Jiang L., Barsan V., Mancusi R. (2022). GD2-CAR T Cell Therapy for H3K27M-Mutated Diffuse Midline Gliomas. Nature.

[35] Monje M., Mahdi J., Majzner R., Yeom K.W., Schultz L.M., Richards R.M., Barsan V., Song K.-W., Kamens J., Baggott C. (2025). Intravenous and Intracranial GD2-CAR T Cells for H3K27M+ Diffuse Midline Gliomas. Nature.

[36] Vitanza N.A., Ronsley R., Choe M., Seidel K., Huang W., Rawlings-Rhea S.D., Beam M., Steinmetzer L., Wilson A.L., Brown C. (2025). Intracerebroventricular B7-H3-Targeting CAR T Cells for Diffuse Intrinsic Pontine Glioma: A Phase 1 Trial. Nat. Med..

[37] Yasinjan F., Xing Y., Geng H., Guo R., Yang L., Liu Z., Wang H. (2023). Immunotherapy: A Promising Approach for Glioma Treatment. Front. Immunol..

[38] Sampath P., Sengupta S., Sengupta S., Junghans R. (2017). IMMU-01. Temozolomide-Resistant Car-T Enhances Glioblastoma Clearance by Concurrent Chemotherapy and Immunotherapy. Neuro-Oncology.

[39] Suryadevara C.M., Desai R., Abel M.L., Riccione K.A., Batich K.A., Shen S.H., Chongsathidkiet P., Gedeon P.C., Elsamadicy A.A., Snyder D.J. (2018). Temozolomide Lymphodepletion Enhances CAR Abundance and Correlates with Antitumor Efficacy against Established Glioblastoma. Oncoimmunology.

[40] Ahmed N., Brawley V., Hegde M., Bielamowicz K., Kalra M., Landi D., Robertson C., Gray T.L., Diouf O., Wakefield A. (2017). HER2-Specific Chimeric Antigen Receptor-Modified Virus-Specific T Cells for Progressive Glioblastoma: A Phase 1 Dose-Escalation Trial. JAMA Oncol..

[41] Vitanza N.A., Johnson A.J., Wilson A.L., Brown C., Yokoyama J.K., Künkele A., Chang C.A., Rawlings-Rhea S., Huang W., Seidel K. (2021). Locoregional Infusion of HER2-Specific CAR T Cells in Children and Young Adults with Recurrent or Refractory CNS Tumors: An Interim Analysis. Nat. Med..

[42] Marcuello C., Lim K., Nisini G., Pokrovsky V.S., Conde J., Ruggeri F.S. (2025). Nanoscale Analysis beyond Imaging by Atomic Force Microscopy: Molecular Perspectives on Oncology and Neurodegeneration. Small Sci..

[43] Andolfi L., Bourkoula E., Migliorini E., Palma A., Pucer A., Skrap M., Scoles G., Beltrami A.P., Cesselli D., Lazzarino M. (2014). Investigation of Adhesion and Mechanical Properties of Human Glioma Cells by Single Cell Force Spectroscopy and Atomic Force Microscopy. PLoS ONE.

[44] Najera J., Rosenberger M.R., Datta M. (2023). Atomic Force Microscopy Methods to Measure Tumor Mechanical Properties. Cancers.

[45] Mount C.W., Majzner R.G., Sundaresh S., Arnold E.P., Kadapakkam M., Haile S., Labanieh L., Hulleman E., Woo P.J., Rietberg S.P. (2018). Potent Antitumor Efficacy of Anti-GD2 CAR T Cells in H3-K27M+ Diffuse Midline Gliomas. Nat. Med..

[46] Quail D.F., Joyce J.A. (2017). The Microenvironmental Landscape of Brain Tumors. Cancer Cell.

[47] Majzner R.G., Theruvath J.L., Nellan A., Heitzeneder S., Cui Y., Mount C.W., Rietberg S.P., Linde M.H., Xu P., Rota C. (2019). CAR T Cells Targeting B7-H3, a Pan-Cancer Antigen, Demonstrate Potent Preclinical Activity Against Pediatric Solid Tumors and Brain Tumors. Clin. Cancer Res..

[48] Henke E., Nandigama R., Ergün S. (2019). Extracellular Matrix in the Tumor Microenvironment and Its Impact on Cancer Therapy. Front. Mol. Biosci..

[49] Mintz A., Gibo D.M., Slagle-Webb B., Christensen N.D., Debinski W. (2002). IL-13Rα2 Is a Glioma-Restricted Receptor for Interleukin-13. Neoplasia.

[50] Fatehi D., Baral T.N., Abulrob A. (2014). In Vivo Imaging of Brain Cancer Using Epidermal Growth Factor Single Domain Antibody Bioconjugated to Near-Infrared Quantum Dots. J. Nanosci. Nanotechnol..

[51] Wang S.S., Davenport A.J., Iliopoulos M., Hughes-Parry H.E., Watson K.A., Arcucci V., Mulazzani M., Eisenstat D.D., Hansford J.R., Cross R.S. (2023). HER2 Chimeric Antigen Receptor T Cell Immunotherapy Is an Effective Treatment for Diffuse Intrinsic Pontine Glioma. Neuro-Oncol. Adv..

[52] Foster J.B., Madsen P.J., Harvey K., Griffin C., Stern A., Patterson L., Joshi N., Dickson C., McManus O., Beaubien E. (2025). Transient mRNA CAR T Cells Targeting GD2 Provide Dose-Adjusted Efficacy against Diffuse Midline Glioma and High Grade Glioma Models. Neuro-Oncology.

[53] Guo Y., Wang X., Zhang C., Chen W., Fu Y., Yu Y., Chen Y., Shao T., Zhang J., Ding G. (2025). Tumor Immunotherapy Targeting B7-H3: From Mechanisms to Clinical Applications. Immunotargets Ther..

[54] Inthanachai T., Boonkrai C., Phakham T., Pisitkun T., Thaiwong R., Chuthaphakdikun V., Sakunrangsit N., Limprasutr V., Chinsuwan T., Hirankarn N. (2025). Novel B7-H3 CAR T Cells Show Potent Antitumor Effects in Glioblastoma: A Preclinical Study. J. Immunother. Cancer.

[55] Nehama D., Ianni N.D., Musio S., Du H., Patané M., Pollo B., Finocchiaro G., Park J.J., Dunn D.E., Edwards D.S. (2019). B7-H3-Redirected Chimeric Antigen Receptor T Cells Target Glioblastoma and Neurospheres. EBioMedicine.

[56] Miller C.D., Lozada J.R., Zorko N.A., Elliott A., Makovec A., Radovich M., Heath E.I., Agarwal N., Mckay R.R., Garje R. (2024). Pan-Cancer Interrogation of B7-H3 (CD276) as an Actionable Therapeutic Target Across Human Malignancies. Cancer Res. Commun..

[57] Mohiuddin E., Wakimoto H. (2021). Extracellular Matrix in Glioblastoma: Opportunities for Emerging Therapeutic Approaches. Am. J. Cancer Res..

[58] Ma Q., Long W., Xing C., Chu J., Luo M., Wang H.Y., Liu Q., Wang R.-F. (2018). Cancer Stem Cells and Immunosuppressive Microenvironment in Glioma. Front. Immunol..

[59] Ma T., Su G., Wu Q., Shen M., Feng X., Zhang Z. (2024). Tumor-Derived Extracellular Vesicles: How They Mediate Glioma Immunosuppression. Mol. Biol. Rep..

[60] Ravi V.M., Neidert N., Will P., Joseph K., Maier J.P., Kückelhaus J., Vollmer L., Goeldner J.M., Behringer S.P., Scherer F. (2022). T-Cell Dysfunction in the Glioblastoma Microenvironment Is Mediated by Myeloid Cells Releasing Interleukin-10. Nat. Commun..

[61] Yin B., Cai Y., Chen L., Li Z., Li X. (2024). Immunosuppressive MDSC and Treg Signatures Predict Prognosis and Therapeutic Response in Glioma. Int. Immunopharmacol..

[62] Flies D.B., Langermann S., Jensen C., Karsdal M.A., Willumsen N. (2023). Regulation of Tumor Immunity and Immunotherapy by the Tumor Collagen Extracellular Matrix. Front. Immunol..

[63] Jiang D., Li Y. (2025). Unraveling the Immunosuppressive Microenvironment of Glioblastoma and Advancements in Treatment. Front. Immunol..

[64] Chen W., Xu L., Guo Z., Zhou M. (2025). Optimizing CAR-T Cell Function in Solid Tumor Microenvironment: Insights from Culture Media Additives. Curr. Res. Transl. Med..

[65] Tsai H.-F., Trubelja A., Shen A.Q., Bao G. (2017). Tumour-on-a-Chip: Microfluidic Models of Tumour Morphology, Growth and Microenvironment. J. R. Soc. Interface.

[66] Li W., Zhou Z., Zhou X., Khoo B.L., Gunawan R., Chin Y.R., Zhang L., Yi C., Guan X., Yang M. (2023). 3D Biomimetic Models to Reconstitute Tumor Microenvironment In Vitro: Spheroids, Organoids, and Tumor-on-a-Chip. Adv. Healthc. Mater..

[67] Liu Z., Zhou J., Yang X., Liu Y., Zou C., Lv W., Chen C., Cheng K.K.-Y., Chen T., Chang L.-J. (2023). Safety and Antitumor Activity of GD2-Specific 4SCAR-T Cells in Patients with Glioblastoma. Mol. Cancer.

[68] Rubin D.B., Al Jarrah A., Li K., LaRose S., Monk A.D., Ali A.B., Spendley L.N., Nikiforow S., Jacobson C., Vaitkevicius H. (2020). Clinical Predictors of Neurotoxicity After Chimeric Antigen Receptor T-Cell Therapy. JAMA Neurol..

[69] Friedman A.R., Tozlu C., Gordillo C.A., Chan H.T., Reshef R., Wesley S.F. (2025). Novel Risk Factors for Predicting Immune Effector Cell-Associated Neurotoxicity Syndrome. medRxiv.

[70] Li J., Chen H., Xu C., Hu M., Li J., Chang W. (2024). Systemic Toxicity of CAR-T Therapy and Potential Monitoring Indicators for Toxicity Prevention. Front. Immunol..

[71] Brudno J.N., Kochenderfer J.N. (2016). Toxicities of Chimeric Antigen Receptor T Cells: Recognition and Management. Blood.

[72] Ma K., Hu P. (2023). Chimeric Antigen Receptor T-Cell Therapy for Glioblastoma. Cancers.

[73] Mahdi J., Dietrich J., Straathof K., Roddie C., Scott B.J., Davidson T.B., Prolo L.M., Batchelor T.T., Campen C.J., Davis K.L. (2023). Tumor Inflammation-Associated Neurotoxicity. Nat. Med..

[74] Logun M., Wang X., Sun Y., Bagley S.J., Li N., Desai A., Zhang D.Y., Nasrallah M.P., Pai E.L.-L., Oner B.S. (2025). Patient-Derived Glioblastoma Organoids as Real-Time Avatars for Assessing Responses to Clinical CAR-T Cell Therapy. Cell Stem Cell.

[75] Khamis Z.I., Sarker D.B., Xue Y., Al-Akkary N., James V.D., Zeng C., Li Y., Sang Q.-X.A. (2023). Modeling Human Brain Tumors and the Microenvironment Using Induced Pluripotent Stem Cells. Cancers.

[76] Vandecandelaere G., Ramapriyan R., Gaffey M., Richardson L.G., Steuart S.J., Tazhibi M., Kalaw A., Grewal E.P., Sun J., Curry W.T. (2024). Pre-Clinical Models for CAR T-Cell Therapy for Glioma. Cells.

[77] Linkous A., Balamatsias D., Snuderl M., Edwards L., Miyaguchi K., Milner T., Reich B., Cohen-Gould L., Storaska A., Nakayama Y. (2019). Modeling Patient-Derived Glioblastoma with Cerebral Organoids. Cell Rep..

[78] Wen J., Liu F., Cheng Q., Weygant N., Liang X., Fan F., Li C., Zhang L., Liu Z. (2023). Applications of Organoid Technology to Brain Tumors. CNS Neurosci. Ther..

[79] Wang G., Zhang Z., Zhong K., Wang Z., Yang N., Tang X., Li H., Lu Q., Wu Z., Yuan B. (2023). CXCL11-Armed Oncolytic Adenoviruses Enhance CAR-T Cell Therapeutic Efficacy and Reprogram Tumor Microenvironment in Glioblastoma. Mol. Ther..

[80] Jacob F., Salinas R.D., Zhang D.Y., Nguyen P.T.T., Schnoll J.G., Wong S.Z.H., Thokala R., Sheikh S., Saxena D., Prokop S. (2020). A Patient-Derived Glioblastoma Organoid Model and Biobank Recapitulates Inter- and Intra-Tumoral Heterogeneity. Cell.

[81] Zarychta J., Kowalczyk A., Marszołek A., Zawitkowska J., Lejman M. (2024). Strategies to Overcome Tumor Microenvironment Immunosuppressive Effect on the Functioning of CAR-T Cells in High-Grade Glioma. Ther. Adv. Med. Oncol..

